# Low Seroprevalence of Brucellosis in Humans and Small Ruminants in the Gambia

**DOI:** 10.1371/journal.pone.0166035

**Published:** 2016-11-08

**Authors:** Eveline A. Germeraad, Lenny Hogerwerf, Tisbeh Faye-Joof, Bart Goossens, Wim van der Hoek, Momodou Jeng, Modou Lamin, Ismaila L. Manneh, Davis Nwakanma, Hendrik I. J. Roest, Arss Secka, Arjan Stegeman, Rita Wegmüller, Marianne A. B. van der Sande, Ousman Secka

**Affiliations:** 1 Centre for Infectious Disease Control, National Institute for Public Health and the Environment, Bilthoven, The Netherlands; 2 Department of Vaccinology, Medical Research Council Unit The Gambia, Banjul, Fajara, The Gambia; 3 Kombo Veterinary Services, Bakau, Gambia; 4 International Trypanotolerance Centre, Banjul, The Gambia; 5 Department of Disease Control and Elimination, Medical Research Council Unit, The Gambia, Banjul, Fajara, The Gambia; 6 Department of Bacteriology and Epidemiology, Central Veterinary Institute, part of Wageningen UR, Lelystad, The Netherlands; 7 Department of Farm Animal Health, Utrecht University, Utrecht, The Netherlands; 8 Medical Research Council (MRC) International Nutrition Group, MRC Unit The Gambia, Keneba, The Gambia; 9 Julius Center for Health Sciences and Primary Care, University Medical Center Utrecht, Utrecht, The Netherlands; Ella Foundation, INDIA

## Abstract

**Background:**

Brucellosis is a worldwide zoonosis with significant impact on rural livelihoods and a potentially underestimated contributor to febrile illnesses. The aim of this study was to estimate the seroprevalence of brucellosis in humans and small ruminants in The Gambia.

**Methods:**

The study was carried out in rural and urban areas. In 12 rural villages in Kiang West district, sera were collected from humans (n = 599) and small ruminants (n = 623) from the same compounds. From lactating small ruminants, milk samples and vaginal swabs were obtained. At the urban study sites, sera were collected from small ruminants (n = 500) from slaughterhouses and livestock markets. Information on possible risk factors for seropositivity was collected through questionnaires. Sera were screened for antibodies against *Brucella* spp. with the Rose Bengal Test, ELISA and Micro Agglutination Test (human sera only). PCR was performed on 10 percent of the milk samples and vaginal swabs from small ruminants.

**Results:**

One human and 14 sheep sera were positive by the Rose Bengal Test. The rest were negative in all serological tests used. The PCR results were all negative.

**Conclusions:**

The results suggest that brucellosis is currently not a generalized problem in humans or small ruminants in The Gambia.

## Introduction

Brucellosis is considered one of the most common globally occurring zoonoses [1]. *Brucella* spp. are intracellular Gram-negative coccobacilli that can infect various species of animals as well as humans [2]. The most common *Brucella* spp. responsible for human brucellosis are *B*. *abortus* (from cattle), *B*. *melitensis* (from goats and sheep), and *B*. *suis* (from pigs). *B*. *melitensis* is the most virulent species for humans [3].

In humans, brucellosis mainly causes febrile illness [2, 4]. As direct person-to-person transmission is extremely rare, animals and their products are considered the only significant source of human brucellosis [5].

In small ruminants, the primary clinical signs of *Brucella* infection are abortion, stillbirths, infertility and decreased milk production. Animal-to-human transmission may occur through direct contact with vaginal and placental fluid and material and aborted fetuses of infected animals, or via consumption of raw milk or unpasteurized dairy products from these animals [5].

Worldwide, brucellosis in both humans and animals is massively underreported, and official numbers constitute only a fraction of the actual occurrence of the disease [4]. However, the prevalence of brucellosis worldwide varies widely across regions. For instance, brucellosis is endemic in Asia and the Middle East and parts of North Africa including Algeria [6]. In other countries, like The Netherlands, bovine brucellosis has been eliminated, being defined by the absence of reported cases for at least five years [4].

For large parts of sub-Saharan Africa, the prevalence of brucellosis is poorly estimated [7]. Particularly in West Africa, information about the prevalence of brucellosis is sparse [6, 7]. Based on a review of seroepidemiological studies, endemic situations are likely in several countries in West Africa, including Ghana, Togo, and Nigeria [6].

In West Africa, human cases of brucellosis have been reported to the World Organisation for Animal Health (OIE) in Burkina Faso and Mali [6]. Caprine or ovine brucellosis have been reported to the OIE in a few West African countries, including Burkina Faso, Niger and Senegal [7].

As the symptoms of human brucellosis are not very specific, clinical brucellosis can be confused with other febrile illnesses, especially, malaria [8]. Misdiagnosis of brucellosis may result in inadequate treatment and unnecessary use of antimalarial medicine [9]. In The Gambia, a major decline in proportions of positive malaria diagnostic test results, hospital admissions and deaths due to malaria over several years until the end of 2007 have been reported [10]. However, numbers of febrile illness have not reduced and may still be considered malaria in the absence of diagnosis to differentiate malaria from other pyrogenic agents [8]. For example, during the 2008 malaria season in Farafenni area in The Gambia, only 11% (24/223) of febrile episodes detected during a 22-week follow-up of a cohort of 800 children were due to malaria [11]. Other causes of febrile illness include invasive bacterial infections such as typhoid fever and emerging or neglected zoonoses such as leptospirosis, Q fever, tick-borne relapsing fever and brucellosis [12–15].

In The Gambia, no recent information about the prevalence of brucellosis in human and small ruminants is available. Such information could assist the differential diagnosis of febrile illness in humans. If brucellosis were prevalent in the region, improved diagnosis could reduce unnecessary use of antimalarial treatment and improve the recovery of individual patients. Therefore, the aim of this study was a) to estimate simultaneously the seroprevalence of brucellosis in humans and small ruminants in a rural area with intensive human-animal contacts, and b) to estimate the seroprevalence of brucellosis in small ruminants transported from different areas in the country to the urban areas for slaughter as potential source of infection for humans, particularly slaughterhouse workers, in the urban areas. In addition, behavioural and environmental risk factors for the transmission of brucellosis were assessed.

## Materials and Methods

### Study sites

**Fig 1:**
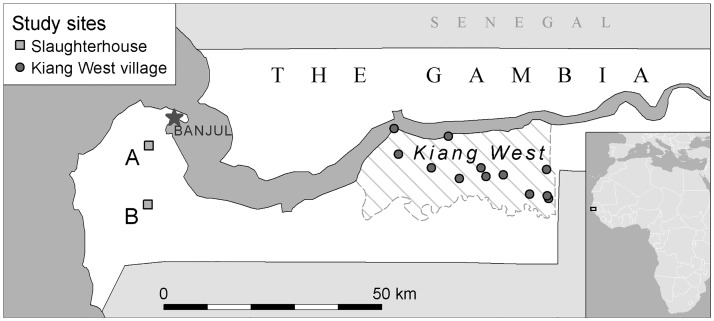
Geographical location of the study sites. Geographical location of The Gambia in West Africa, with the capital city Banjul, the urban study sites Abuko (indicated by A) and Brikama (indicated by B) and the rural study area Kiang West (indicated by a striped shaded area) with the 12 study villages.

The study was performed in 12 villages in Kiang West district, a rural area in the Lower River Region (LRR) and in Abuko and Brikama, two urban sampling sites in the West Coast Region (WCR) of The Gambia (Fig 1). Kiang West, situated approximately 130 km by road east of the capital Banjul, is a rural area with 34 small villages with an estimated population of 15000 people, and 8000 goats and 1600 sheep. Both the urban study sites were slaughterhouses and their associated livestock markets. The first urban study site was the central slaughterhouse with associated livestock market in Abuko, located in Kombo North district of WCR, where around 30 small ruminants are slaughtered daily. The second urban study site was the livestock market of Brikama, located in Kombo Central district (WCR), where around 20 small ruminants are slaughtered daily in the associated slaughterhouse.

### Study period

The study was carried out from September to December 2014, just after the rainy season and lambing season. This period was chosen for two reasons. First, bacterial shedding of *Brucella* spp. by small ruminants was expected to peak during lambing season (through abortions), leading to animal and human infections and boosting the immune system to elevate the antibody levels that could be best detected by serological testing after the lambing season. Second, at the end of the rainy season, small ruminants are flocked together in a controlled way to avoid crop damage, and therefore kept longer in the compound, making sampling easier. Traditionally and for safe-guarding, owners keep their small ruminants at night either in their house or close to the house leading to higher chances of exposure of humans to infected animals.

### Study population

In rural areas of The Gambia, humans and small ruminants typically live closely together in compounds, a gated community where a few families live together. Households commonly own a few small ruminants, which are used for religious celebrations, serve as savings or emergency cash or provide meat or milk [16]. The most common small ruminant breeds in The Gambia are the West African Dwarf goats and Djallonke sheep. Sahelian long-legged sheep and goats, originally from Senegal, sometimes crossed with the West African dwarf breeds, are also common [16]. Small ruminants traded at live animal markets in the urban areas of The Gambia originate from a wider catchment area, including Senegal.

### Sample size

The target sample size was based on an expected prevalence of 10% [5]. With a relative precision of 3.5% and a confidence level at 95%, we needed to include 283 individuals [17]. However, some clustering of the seropositive individuals was expected and therefore the sample size was adjusted with a design effect of 2 [18]. The total target sample size was therefore set at 600 for human and small ruminants (in rural and urban area) each. With this sample size, accounting for clustering, a seroprevalence as low as 1% is expected to be detectable.

### Sampling strategy

Kiang West was selected because of its abundance of small ruminants as well as the existing infrastructure related to human health through the Medical Research Council (MRC), and animal health through the International Trypanotolerance Centre (ITC) both based in the Village of Keneba, more or less central in the sampling area.

The 12 villages were selected based on the number of inhabitants and small ruminants (aiming for sufficient numbers) and their topographic location in Kiang West (aiming for variation). Villages were included in the study with permission from the Alkalo (chief of the village).

In each selected village, compounds with a minimum of 5 small ruminants and 5 adults were listed, using the Kiang West Demographic Surveillance System of MRC [19], the livestock census of 2013 of PROGEBE [20], and field observations. From this list of compounds meeting the inclusion criteria, compounds were randomly selected for each village to be potentially included in the study. During field visits, a final selection of the first 10 eligible compounds listed was made, based on the actual presence of adults and small ruminants. When it was not possible to select 10 compounds with at least 5 small ruminants and 5 adults, additional compounds (with the highest numbers of small ruminants and adults) were included in the study to reach the target sample size of approximately 50 adults and 50 small ruminants per village.

For each compound, all the individuals aged 18 years or older, healthy, and not participating in another study were listed using the Kiang West Demographic Surveillance System of MRC [19]. From the list, up to 15 individuals were randomly selected per compound, resulting in a list of potential participants. During the village visits, the potential participants were visited and included in or excluded from the study. Reasons for exclusion were refusal to participate, not living in the compound anymore, being too far away at the time of the field visit, and not being fit to participate. After exclusion of a potential participant, the next person on the list was contacted until on average 5 adults were included in each compound.

Small ruminants were selected from the same compounds as the human participants. In every compound, all present healthy small ruminants of at least 6 months were identified. Around five of these animals were selected ‘randomly’, however, because there were less sheep than goats sometimes sheep were preferred above goats.

In the livestock markets in Abuko and Brikama and the slaughterhouse in Abuko, only healthy animals of at least 6 months of age, identified by dentition as described earlier [16] were included in the study. Sampling was based on convenience until 250 animals were included in each location.

### Ethical approval

For the part related to human participants, the Joint Gambia Government—MRC Unit Ethics Committee approved the proposal (SCC1398v2). Written informed consent was obtained from all participants prior to participation. The veterinary part of the study described in this manuscript was conducted in compliance with legislation on animal experimentation and practicing veterinary medicine of both The Netherlands and The Gambia. The study is not an animal experiment, but an epidemiological study in the field using common sampling methods for routine diagnostic purposes. According to Dutch legislation, such studies do not need approval from an animal ethics committee but they need to be performed according to the Dutch Veterinary Practice Act. Small ruminants were included in the study after obtaining permission from the owner. The village Alkalos, compound heads, and the animal owners gave verbal permission to conduct the study on the study sites. Managers of Abuko Central Abattoir and Livestock market and Brikama Livestock market also permitted and facilitated the sampling processes.

### Interviews, questionnaires and sampling

General information about milk consumption and milk treatment before consumption by the villagers and information about presence of special grazing areas was collected in an interview with the chief of the village, the Alkalo.

For the human participants, once written informed consent was obtained, a questionnaire was used to collect information on possible risk factors and confounders, including sex, age, ethnic group, socio-economic status, animal contact by occupation and raw milk consumption. All information was anonymized.

For small ruminant flock owners and caretakers in the villages in Kiang West, a questionnaire was used to collect information on animals’ reproductive history, risk factors for infection and hygiene after animal contact. For each animal, information on species, breed, sex and age was registered based on observation.

Human or animal blood samples were collected from the cephalic or the jugular vein, respectively, in 5 mL Vacutainer serum tubes (Becton, Dickinson and Company, New Jersey, US), using 21G x 1.5 and 20G x 1.5 Precision Glide Multi-sample needles, respectively (Becton, Dickinson and Company, Franklin Lakes, New Jersey, US).

Milk samples and vaginal swabs were obtained from lactating goats and sheep in Kiang West only. The milk was collected in a 10 mL plastic tube. The teat was cleaned with alcohol-soaked cotton balls before sampling, and the first few streams of milk were discarded. Before taking a vaginal swab the vulva was cleaned with alcohol-soaked cotton balls. A dry and sterile cotton-tipped swab (Copan Flock Technologies Srl., Brescia, Italy) was used to collect the sample.

Within a few hours after sampling, blood samples were centrifuged for 15 minutes at a speed of 1500 *g* [21]. The sera were then divided into different aliquots. Sera, vaginal swabs and milk samples were stored at -20°C until used.

### Diagnostic tests

All human sera were subjected to three different assays: Rose Bengal Test (RBT), Microagglutination test (MAT) and “SERION ELISA classic Brucella IgG” (Institut Virion\Serion GmbH, Würzburg, Germany) in the Serology Laboratory of the MRC Unit, The Gambia.

All animal sera were subjected to RBT and “PrioCHECK Brucella Antibody 2.0 ELISA kit” (Life Technologies Europe BV, Bleiswijk, The Netherlands) in the ITC laboratory at Kerr Serign, The Gambia. Animal sera were not tested with the MAT because the MAT has not been validated for small ruminant sera.

In the laboratory of the Central Veterinary Institute (CVI) (Wageningen UR, Lelystad, The Netherlands) inter-laboratory comparison was performed for ELISA (human and animal sera), RBT (human and animal sera) and MAT (human sera only). For this, a selection of human, goat and sheep sera was made from compounds with one or more positive subject(s), with an additional random selection of seronegative sera to reach a 10% sample. At the CVI, a Complement Fixation Test (CFT) was used additionally for final classification in case of any discrepancy between results of the RBT, ELISA, and MAT.

For the RBT, MAT and CFT, CVI protocols were used. The protocols for RBT and CFT are based on the guidelines described in the OIE Terrestrial Manual (2012) [22]. The RBT, with *B*. *abortus* antigen (IDEXX, the Netherlands), was considered negative when there was no agglutination after 4 minutes and positive when there was any visible reaction.

The MAT was performed with a commercial *B*. *abortus* antigen (Symbiotics Europe, France) and positive and negative serum controls (CVI, Lelystad, The Netherlands). A working dilution of the antigen, with 0.5% phenol-saline solution was used. In 96-well U-shaped microplates, the working dilution of the antigen and (control) serum were diluted from 1:7.5 to three more two-fold dilutions. The 25% antigen control was prepared for each test by diluting 25 μL antigen with 75 μL phenol/saline solution. The MAT was performed by incubating the sera at 37°C +/- 1°C for 21 +/- 1 hours covered with a plastic seal. In the MAT, sera were considered positive with a titre equivalent to 30 I.U./mL (1:15) or more [22].

In the CFT (with in-house produced antigen derived from *B*. *abortus* strain 99), sera producing a titre equivalent to 20 ICFTU (international complement fixation test units) per mL or more were considered to be positive [22].

The ELISA analyses were carried out according to the manufacturer’s instructions, where the cut-off values are also described. Goat and sheep have different cut-off values: sample to positive ratios <30% for sheep and <40% for goats were classified as negative.

Following serology, 15 vaginal swabs and milk samples, representing 10% of the total sample, were selected for PCR analysis at CVI. The selection was based on the positive serological results; every vaginal swab and milk sample from animals in a compound with one or more RBT positive individual animal(s) was selected for PCR (with addition of some randomly selected samples to complete the 10% sample). Milk samples and swabs originated from the same animals. DNA was extracted using the NucliSens easyMag extractor (bioMérieux, Marcy l’Etoile, France) as described previously [23]. The PCR was performed as described previously [24].

All figures were produced in R version 3.1.0 [25].

## Results

In Kiang West, human serum samples were obtained from 599 humans in 125 compounds and animal serum samples were obtained from 623 small ruminants (494 goats and 129 sheep) in 122 compounds. In the urban study sites (Abuko and Brikama), 500 small ruminants (250 goats and 250 sheep) were sampled (Table 1).

**Table 1:** Number of compounds, goats, sheep and humans included in the investigation at different study sites.

		No. compounds	No. goats	No. sheep	No. human
**Villages in Kiang West**	Dumbuto	11	48	4	50
	Jali	10	33	17	50
	Janneh Kunda	11	41	15	50
	Jiffarong	10	40	10	50
	Kantong Kunda	11	44	15	50
	Kemoto	13	29	6	47
	Keneba	11	50	7	50
	Kuli Kunda	10	46	4	52
	Manduar	10	41	17	50
	Niorro Jattaba	10	36	15	50
	Sankandi	10	37	16	49
	Tankular	10	49	3	51
	Total	127	494	129	599
**Abuko**		NA^a^	199	216	ND^b^
**Brikama**		NA^a^	51	34	ND^b^
**Grand total**		**127**	**744**	**379**	**599**

^a^No compounds, slaughterhouses or livestock markets

^b^No human participants included in Abuko and Brikama

### Human study population and risk factors

In Kiang West, the most common ethnicity is Mandinka (78.4%), followed by Fula (17.4%) and Jola (2.2%). The population consists of 59.4% females and 40.6% males. The demographic background of the study population is comparable to the total human population of Kiang West. Exceptions are a higher percentage of Mandinka ethnicity (89.6% versus 78.4%) and a lower percentage of ≥80 years old people (1.0% versus 3.1%) among study participants (Table 2).

**Table 2:** Overview of sex, ethnicity and age of the study participants and of the population in Kiang West.

		Study Participants		Kiang West population^a^	
**Sex**	Female	403	(67.3%)	3986	(59.4%)
	Male	196	(32.7%)	2721	(40.6%)
**Ethnicity**	Mandinka	537	(89.6%)	5260	(78.4%)
	Fula	55	(9.2%)	1167	(17.4%)
	Jola	2	(0.3%)	146	(2.2%)
	Other	5	(0.8%)	134	(2.0%)
**Age**	18–19	54	(9.0%)	645	(9.6%)
	20–29	123	(20.5%)	1743	(26.0%)
	30–39	125	(20.9%)	1210	(18.0%)
	40–49	110	(18.4%)	992	(14.8%)
	50–59	80	(13.4%)	901	(13.4%)
	60–69	76	(12.7%)	666	(9.9%)
	70–79	25	(4.2%)	344	(5.1%)
	80>	6	(1.0%)	206	(3.1%)
**Total number of individuals**		**599**		**6707**	

^a^ Data from the Kiang West Demographic Surveillance System of MRC^15^.

The study participants reported several potential risk factors for brucellosis. For example, 56.3% reported to be animal farmers. Consumption of raw cattle milk during the lifetime was most common (95.8%). Also, raw sheep milk (10.2%) and goat milk (43.7%) were consumed during lifetime, especially during childhood when the children herd the small ruminants. Only a small fraction of the study participants had consumed sheep milk (0.2%) or goat milk (0.8%) in the preceding 14 days. Practices of flock owners with respect to hygienic measures after abortion and contact with the small ruminants showed no clear pattern. After contact with small ruminants, 42.4% of the flock owners indicated not to wash their hands usually, while 18.5% indicated to usually wash their hands with water and soap.

### Small ruminant study population and risk factors

In Kiang West, goats are more common than sheep, which was reflected in the number of serum samples collected: 129 sheep and 494 goats. From the lactating animals, 153 vaginal swabs and 151 milk samples were collected. In the urban study sites, sera were collected from 250 goats and 250 sheep.

Over half of the flocks (55.6%) in Kiang West had at least one abortion in the previous year. Almost all flocks were free ranged during the dry season (99.3%), allowing direct contact between the small ruminants of different flocks, with sheep and goats usually intermixing. During the rainy season, 62.3% of the flocks were tethered for grazing or were herded in the afternoon to graze, in larger flocks of different households together, also allowing contact with other flocks.

### Human serology

**Fig 2:**
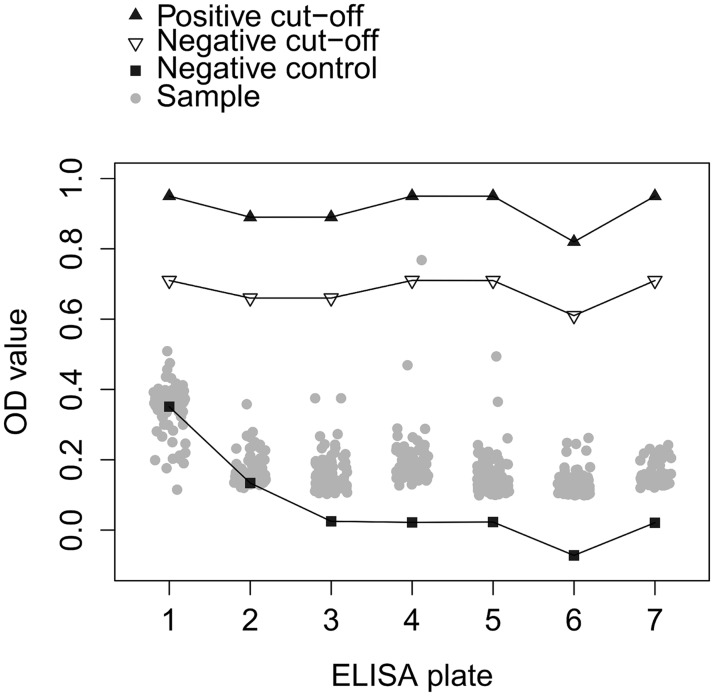
ELISA results for human sera. Optical density values measured by SERION ELISA classic Brucella IgG for human sera (n = 599), including the OD value of the positive cut-off, negative cut-off and the negative control for each ELISA plate. Each grey dot in the figure refers to one serum sample. When the OD-value of the sample is above the positive cut-off, the sample is positive; when it is below the negative cut-off, the sample is negative. When the OD-value of the sample is in-between the negative and positive cut-off, the sample is a borderline and a definitive interpretation of the result is not possible.

Only one human tested positive by RBT. Four human sera had a slight reaction by MAT of 15 IU. The one person that was positive for RBT also had one of the four MAT reactive samples. Another person tested ‘borderline’ by ELISA (below the positive cut-off but above the negative cut-off). All others were negative by ELISA. The human serological results are summarized in Table 3. The Optical Density values (OD-values) of the ELISA are shown in Fig 2.

**Table 3:** Serological results of humans, sheep and goats in Kiang West, Abuko and Brikama.

		No. tested	No. pos. RBT	No. pos. MAT	No. pos. ELISA
**Humans**	Kiang West	599	1	0^a^	0^b^
**Goats**	Kiang West	494	0	-	0
	Abuko and Brikama	250	0	-	0
	*Total*	744	0	-	0
**Sheep**	Kiang West	129	10	-	0
	Abuko and Brikama	250	4	-	0
	*Total*	379	14	-	0

^a^ Four human samples had a slight reaction by MAT (15 IU) but following OIE guidelines these samples are considered negative. MAT was not performed with goat and sheep sera.

^b^ One human sample had a borderline ELISA result (below the positive cut-off but above the negative cut-off)

### Small ruminant serology and PCR

**Fig 3:**
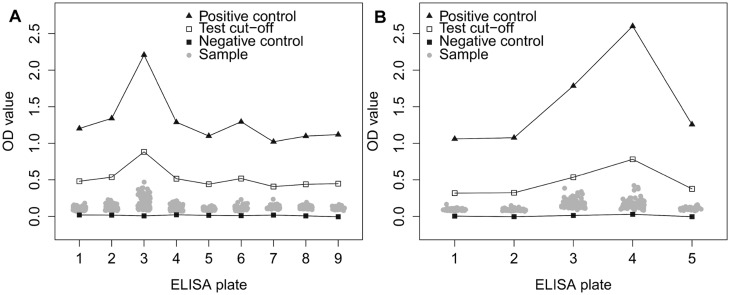
ELISA results for goat and sheep sera. Optical density values obtained using PrioCHECK Brucella Antibody 2.0 ELISA kit for small ruminant sera, including the OD value of the positive cut-off, negative cut-off and the negative control for each ELISA plate. Each grey dot in the figure refers to one serum sample. When the OD-value of the sample is above the test cut-off, the sample is positive; when it is below the test cut-off, the sample is negative. The results for goat sera (n = 744) and sheep sera (n = 379) are displayed in different panels because they have a different cut-off value. Results for goat sera, with a test cut-off of 40% of the positive control, are shown in panel A. Results for sheep sera, with a test cut-off of 30% of the positive control, are shown in panel B.

All small ruminants in the study tested negative by ELISA, and all goats tested negative by RBT. In Kiang West, 10 sheep tested positive by RBT. In the urban study sites, four sheep tested positive by RBT. All vaginal swabs (n = 15) and milk samples (n = 15) tested by PCR were negative for *Brucella* spp. DNA. The veterinary serological results are summarized in Table 3. The OD values of the ELISA for goats and sheep are shown in Fig 3A and 3B. Goats and sheep are plotted in different figures because they have a different cut-off value for this version of ELISA.

### Validation of serological tests

Additional testing by CFT, for final classification in case of discrepancies, was performed in 14 sheep sera and 5 human sera. This concerned 14 sheep sera with positive RBT and negative ELISA, one human serum with positive RBT, negative ELISA and a slight reaction on the MAT, three human sera with only a slight reaction on the MAT, and one human serum with a borderline outcome in the ELISA. In all instances, CFT results were negative.

The inter-laboratory comparability between the laboratories in The Gambia and The Netherlands was good. Based on the standard test cut-offs for positive and negative results, agreement was 100% for all tests, with the exception of RBT for animal sera (64 negative sera in agreement, 11 positive sera in agreement, 5 not in agreement, Cohen's Kappa = 0.78 [17]).

## Discussion

In this study we were unable to find antibodies against *Brucella* spp. in humans as well as small ruminants in a rural area in The Gambia. This is remarkable, as brucellosis is considered the world’s most common bacterial zoonosis [6]. Possible risk factors for the transmission of brucellosis from small ruminants to humans, such as consumption of raw milk and close contact with domestic farm animals, were prevalent in the study area. Previously, in other countries of Western Africa, varying *Brucella* seroprevalence in humans, small ruminants and cattle have been reported. In Nigeria, reported seroprevalences ranges between 0–50% in cattle, 0–76% in small ruminants and 0–74% in humans [5]. In neighbouring Ivory Coast in 2005–2009 a prevalence of 4.6% was reported in the sera of 995 cows [26]. A study in Togo in 2011–2012 showed a seroprevalence of 2.6% in humans and 16.5% in cattle, while no antibodies were detected in small ruminants [1]. In The Gambia, in 2001–2003, seropositive cattle have been reported with a prevalence of 1.1% [27]. One could question whether the 1.1% seroprevalence in cattle is much different from the result found in the present study. Given the negative serological results, the estimated maximum seroprevalence in small ruminants and humans is around 1% [17].

One explanation for the low prevalence in the present study is that we might have missed humans and small ruminants with previous exposure to *Brucella* because brucellosis can occur in clusters [28]. While some clustering was taken into account when determining the sample size, brucellosis can occur more clustered than anticipated. Therefore, future investigations could target herds with reported abortions or reproductive disorders. To better assess the presence of Brucellosis in humans in The Gambia, febrile patients can be tested with serological or molecular diagnostic tests. This might give a better insight in the cause of the fever. Such a study would have to consider additional potential causes of febrile illness, including zoonotic bacterial diseases, such as Q-fever or leptospirosis.

The results of this study show some discrepancy between ELISA and RBT, with fourteen sheep and one human testing positive in RBT but negative in ELISA. The RBT positive sheep and human originated from various study sites and appeared unrelated, with exception of two sheep that originated from the same flock. Ten of the RBT positive sheep originated from the villages in Kiang West, from mixed flocks with goats. In general, the agreement between ELISA and RBT is good for the negative results (96.6%; CI 95.7–97.4) but is lower for the positive results (52.2%; CI 41.9–62.5) [26]. ELISA is generally considered to be more sensitive than RBT [26], therefore, it is surprising that the RBT positive samples were negative in ELISA. The positive RBT sera from our study were also all negative in CFT. Therefore, the positive results of the RBT may have been false-positive. Inconsistency between the RBT, ELISA and CFT tests was also reported by Dean et al. (2013) [1]. However, it is unclear if the positive RBT sera in the study of Dean et al. (2013) pertain to sheep or also goats. To our knowledge, there is no information that sheep have more chance than goats for false-positive RBT results.

## Conclusions

The hypothesis that brucellosis is endemic in the investigated rural and urban areas of The Gambia could not be confirmed in the present study. Seropositivity for *Brucella* spp. was found in only a very small percentage of humans and small ruminants in rural and urban Gambian sites, although risk factors for obtaining *Brucella* infection were present.

## Supporting Information

S1 Table Animal and Flock data. Sheet A: data on all included animals. Sheet B: data on the included small ruminant flocks. Sheet C: explanatory Data Dictionary.
(XLSX)
